# Assessing Olfactory Acuity in Primary Ciliary Dyskinesia with the *RSPH4A* Founder Mutation

**DOI:** 10.3390/jcm14103612

**Published:** 2025-05-21

**Authors:** Miguel A. De Jesús, Wilfredo De Jesús-Rojas

**Affiliations:** Department of Pediatrics and Basic Science, Ponce Health Sciences University, Ponce, PR 00716, USA; mdejesus21@stu.psm.edu

**Keywords:** primary ciliary dyskinesia, *RSPH4A* founder mutation, olfactory function, anosmia, Puerto Rico

## Abstract

**Background/Objectives**: Primary Ciliary Dyskinesia (PCD) is a rare genetic condition characterized by compromised mucociliary clearance and chronic respiratory manifestations. Anosmia, or the loss of smell, is a lesser-known but clinically relevant symptom that can significantly impact patient safety, nutritional status, and the overall quality of life. The *RSPH4A* (c.921+3_921+6delAAGT) founder mutation is highly prevalent among Puerto Rican individuals with PCD and may carry distinct phenotypic implications. This study aimed to evaluate olfactory function in Puerto Rican PCD patients with this mutation using the Brief Smell Identification Test (BSIT^®^) and to assess associations with age and sex. **Methods**: We conducted a case–control study involving 30 participants, including 15 PCD patients with genetically confirmed *RSPH4A* mutations and 15 age- and sex-matched healthy controls. All participants completed the BSIT, and BSIT scores were compared by diagnosis, sex, and age. **Results**: PCD patients had significantly lower BSIT scores than controls (*p* = 0.0015). When stratified by sex, both male (*p* = 0.0289) and female (*p* = 0.0178) PCD patients demonstrated significantly lower BSIT scores compared to their respective healthy counterparts. Regression analysis showed a significant inverse correlation between age and BSIT score in the PCD group (r^2^ = 0.2873; *p* = 0.0395), while no such relationship was observed in controls (r^2^ = 0.0096; *p* = 0.7283). Among PCD patients, age-related decline in olfactory function was more pronounced in females (r^2^ = 0.71; *p* = 0.005) than in males (r^2^ = 0.31; *p* = 0.25). **Conclusions**: These findings demonstrate that the *RSPH4A* founder mutation is associated with measurable olfactory impairment in PCD patients, particularly in females and with advancing age. The routine assessment of olfactory function should be considered in the clinical evaluation of patients with PCD, as anosmia may represent a key phenotypic feature and contribute to disease burden.

## 1. Introduction

Primary Ciliary Dyskinesia (PCD) is an autosomal recessive disorder characterized by impaired ciliary motility, which results in defective mucociliary clearance. Clinically, PCD presents with chronic respiratory tract infections, persistent sinusitis, middle ear disease, and subfertility due to impaired ciliary movement in various tissues [1,2]. Despite its heterogeneous clinical manifestations, olfactory dysfunction has received relatively limited attention in the literature, despite its critical implications for the quality of life [3,4]. Olfactory loss in PCD patients is frequently overlooked in clinical assessments, even though it can negatively affect essential aspects of life such as nutrition, safety, and psychological well-being. Patients with anosmia are unable to detect environmental hazards such as smoke or gas leaks and often experience impaired flavor perception, which may lead to reduced appetite, weight loss, and malnutrition [5,6]. These sensory deficits can lead to psychological effects, including social withdrawal, anxiety, and decreased life satisfaction [7]. The etiology of olfactory dysfunction in PCD is likely multifactorial. Chronic rhinosinusitis, which is prevalent among PCD patients, contributes to mucosal edema and the obstruction of olfactory clefts [6]. Additionally, defective motile cilia in the olfactory epithelium may impair odorant access to sensory neurons [6,8]. Previous studies have shown that *RSPH4A* encodes a radial spoke head protein essential for the structural integrity of the central apparatus of motile cilia [9]. The c.921+3_921+6delAAGT mutation, located in a conserved intronic splice site, leads to aberrant mRNA splicing and results in the absence of the functional RSPH4A protein [9,10]. This mutation is particularly prevalent in the Puerto Rican population, where it has been identified as a founder mutation and is commonly associated with ultrastructural ciliary abnormalities, such as the absence or disorganization of the central pair microtubules (9 + 0 pattern) in airway epithelial cells [11]. Recent genetic studies have established this mutation as a major contributor to the molecular diagnosis of PCD in Puerto Rico [12,13], providing an opportunity to explore genotype–phenotype correlations. Despite its prevalence, the phenotypic expression of this mutation, particularly regarding olfactory function, remains underexplored. This study aims to bridge this knowledge gap by assessing olfactory function in Puerto Rican individuals with PCD due to the *RSPH4A* mutation, using the Brief Smell Identification Test (BSIT^®^) (Sensonics International, Haddon Heights, NJ, USA) [14,15]. We also aim to understand how age and sex may influence olfactory outcomes within this cohort.

## 2. Methods

### 2.1. Study Design and Participants

We conducted a cross-sectional case–control study aimed at evaluating olfactory function in individuals diagnosed with PCD who are homozygous or compound heterozygous for the Puerto Rican founder mutation in the *RSPH4A* gene (c.921+3_921+6delAAGT). This specific mutation has been previously characterized as a common cause of PCD among Puerto Rican individuals and is associated with central apparatus defects in motile respiratory cilia [11]. A total of 30 participants were enrolled in this study, comprising 15 genetically confirmed PCD patients and 15 healthy controls matched by age and sex. The median age of all participants was 37 years, with an interquartile range (IQR) of 40 years; since subjects were age-matched between groups, the same median and IQR apply to each subgroup. Participants in the PCD group were identified through the accredited Puerto Rico PCD Center, which collects clinical, genetic, and demographic information on individuals diagnosed with PCD across the island. Healthy controls were recruited from the general community through outreach at local clinics and health fairs and were screened to exclude any chronic respiratory or sinonasal conditions. Inclusion criteria for both groups included being over the age of 10, the ability to comply with olfactory testing, and no history of acute upper respiratory tract infection, active allergic rhinitis, or sinonasal surgery in the three months preceding the assessment. Additional exclusion criteria included the use of medications that may affect olfactory function (e.g., intranasal steroids or antihistamines) within two weeks prior to testing, as well as neurological or cognitive impairments that could compromise test validity. All participants or their legal guardians provided written informed consent prior to participation, in accordance with the ethical principles outlined in the Declaration of Helsinki. The study protocol was reviewed and approved by the Institutional Review Board (IRB) of Ponce Health Sciences University under protocol number 2307158336, with approval granted on 5 October 2023.

### 2.2. Olfactory Function Testing

Olfactory performance was assessed using the Brief Smell Identification Test (BSIT), a validated and standardized tool commonly used in epidemiological and clinical research to screen for olfactory dysfunction. The BSIT is an abbreviated version of the University of Pennsylvania Smell Identification Test (UPSIT) and maintains strong psychometric properties despite its shorter format. It is particularly suited for use in outpatient and community-based studies due to its rapid administration and minimal resource requirements.

The BSIT consists of 12 odorant stimuli, each encapsulated in a scratch-and-sniff label embedded on a test booklet page. The odorants are microencapsulated and become volatile when scratched, releasing scent molecules that participants can detect and identify. The selected odors include familiar scents such as banana, gasoline, smoke, cinnamon, lemon, and soap, designed to be recognizable across a broad demographic. Each item is accompanied by a forced-choice format, offering four written response options, one of which correctly matches the scent. Participants were instructed to use a provided included pencil to gently scratch the odorant strip. Immediately after releasing the scent, they were asked to select the option that best matched their perception of the odor. No time limits were imposed, but participants were encouraged to rely on their initial perception. If uncertain, they were advised to make their best guess, as the test does not include a “no response” option. Each correctly identified odorant was scored as 1 point, while incorrect responses received 0 points, for a maximum possible score of 12. The interpretation of BSIT scores followed established cutoffs: 0–5: anosmia (complete loss of smell); 6–8: hyposmia (reduced olfactory function); 9–12: normosmia (normal olfactory function)

The BSIT has been validated across diverse populations and shows high test–retest reliability and strong correlation with the full UPSIT (r > 0.9). It is also relatively insensitive to language or cultural background due to its multiple-choice format and use of universally familiar odors. To minimize confounding factors, olfactory testing was conducted in a scent-free environment under standardized conditions. Participants were asked to abstain from food, beverages (other than water), tobacco use, and the application of scented products for at least 30 min prior to testing. While participants were screened to exclude the recent use of corticosteroids and other known olfactory confounders, we acknowledge that prior environmental exposures or therapeutic history may have residual effects on olfactory performance. Testing was conducted by trained personnel following a scripted protocol to ensure consistency and reproducibility across participants.

### 2.3. Statistical Analysis

Descriptive statistics, including median and interquartile ranges (IQRs), were calculated to summarize the BSIT score data, providing measures of central tendency and dispersion. The normality of the dataset was evaluated using the Kolmogorov–Smirnov test. To compare BSIT scores between PCD patients and healthy controls, as well as between male and female subgroups, the paired *t*-test was applied. Age-related trends in BSIT scores were analyzed using simple linear regression to assess whether a linear relationship existed between age and olfactory performance. This approach allowed us to determine the presence of progressive decline across the age spectrum within and between groups. A two-tailed significance level of α = 0.05 was established for all analyses. *p*-values less than 0.05 were considered statistically significant. All statistical analyses were performed using GraphPad Prism version 10.4.1 for macOS (GraphPad Software, San Diego, CA, USA; www.graphpad.com, accessed in 4 February 2025).

## 3. Results

**Figure 1:**
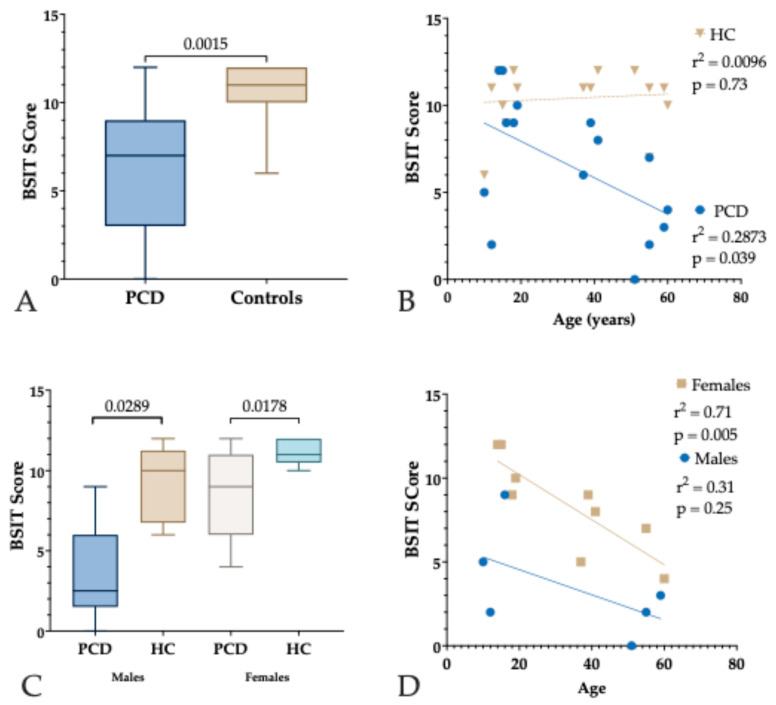
A graphical summary of BSIT (Brief Smell Identification Test) scores in patients with Primary Ciliary Dyskinesia (PCD) carrying the *RSPH4A* founder mutation compared to healthy controls (HC). (**A**) A box-and-whisker plot comparing BSIT scores between PCD patients and healthy controls, showing significantly reduced olfactory function in the PCD group (*p* = 0.0015). (**B**) A scatter plot of BSIT scores by age in both groups. A significant inverse correlation is observed in the PCD group (r^2^ = 0.2873; *p* = 0.0395), whereas no significant correlation is present in the control group (r^2^ = 0.0096; *p* = 0.7283). (**C**) A sex-stratified comparison of BSIT scores in the PCD and control groups. Both male and female PCD patients had significantly lower scores compared to their respective controls (*p* = 0.0289 for males; *p* = 0.0178 for females). (**D**) Age-related trends in BSIT scores within the PCD group, stratified by sex (*n* = 9 females; *n* = 6 males). A significant negative correlation between age and BSIT score is observed in females (r^2^ = 0.71; *p* = 0.005), while the trend in males is non-significant (r^2^ = 0.31; *p* = 0.25).

PCD patients demonstrated significantly reduced olfactory function compared to healthy controls. The median BSIT score for the PCD group was 7, IQR:6, indicating predominantly hyposmic to anosmic performance, while the control group had a median score of 11, IQR:2, consistent with normosmia. Statistical comparison using a paired *t*-test revealed a highly significant difference between the two groups (*p* < 0.0015), as illustrated in Figure 1A. To explore the effects of age on olfactory function, BSIT scores were plotted against participant age in both groups (Figure 1B). In the PCD group, linear regression analysis revealed a statistically significant inverse correlation between age and BSIT score (r^2^ = 0.2873; *p* = 0.039), suggesting that olfactory function declines progressively with age in individuals carrying the *RSPH4A* founder mutation. In contrast, the healthy control group showed no significant association between age and BSIT scores (r^2^ = 0.0096; *p* = 0.73), indicating that olfactory function remains relatively stable across age in individuals without PCD.

**Sex-stratified analyses** provided further insights into potential sex-related differences in olfactory outcomes (Figure 1C). Among males, individuals with PCD had significantly lower BSIT scores compared to healthy male controls (*p* = 0.0289). Similarly, female PCD patients also showed significantly reduced BSIT scores compared to their healthy female counterparts (*p* = 0.0178). A further exploratory analysis stratified PCD patients by both age and sex revealed distinct patterns in the relationship between age and olfactory function (Figure 1D). Among females with PCD, there was a strong and statistically significant inverse correlation between age and BSIT score (r^2^ = 0.71; *p* = 0.005), indicating a progressive decline in olfactory function with advancing age. In contrast, male PCD patients also demonstrated a negative trend, but the correlation was weaker and did not reach statistical significance (r^2^ = 0.31; *p* = 0.25).

## 4. Discussion

This study adds new evidence to the emerging recognition of olfactory dysfunction as a significant clinical manifestation of PCD, particularly among Puerto Rican patients carrying the *RSPH4A* founder mutation. Our results showed that patients with PCD had significantly lower BSIT scores than age- and sex-matched healthy controls, confirming that olfactory dysfunction is not only present but pronounced in this genetic subtype.

Previous studies have reported olfactory impairment in patients with various ciliopathies, including PCD, although most did not focus on specific genotypes. Uytingco et al. highlighted that dysfunction in motile cilia contributes to impaired odorant access and transport to olfactory receptors, impacting olfactory perception [6]. This is consistent with our findings, where the *RSPH4A* mutation—known to disrupt axonemal architecture—was associated with reduced BSIT performance. Our findings are in line with the literature showing that chronic rhinosinusitis, which is ubiquitous in PCD patients, can result in edema and the inflammation of the olfactory clefts, further compounding olfactory impairment [8,16]. However, few studies have quantitatively assessed smell loss in PCD patients using validated tools such as the BSIT. Our use of this rapid, standardized test highlights its utility in identifying anosmia and hyposmia in a clinical setting [14,15].

Interestingly, we found that both male and female PCD patients demonstrated significantly reduced BSIT scores compared to their respective healthy controls, with females exhibiting a more pronounced age-related decline in olfactory function. This is particularly noteworthy given the well-established higher baseline olfactory sensitivity in women [17] and at first glance may appear counterintuitive. However, our findings suggest that female patients may experience a greater relative decline in olfaction, possibly due to increased susceptibility to chronic airway inflammation, hormonal influences on olfactory neuroepithelium, or sex-specific effects of the *RSPH4A* founder mutation on ciliary function. Notably, in our stratified regression analysis, females with PCD exhibited a strong and statistically significant inverse correlation between age and olfactory performance, a pattern not observed in males. Sex differences in olfactory impairment have also been reported in other respiratory and neurodegenerative conditions, yet the underlying biological mechanisms remain unclear and warrant further investigation in the context of PCD [18].

The age-dependent decline in BSIT scores among PCD patients, but not in healthy controls, supports the hypothesis that olfactory dysfunction in this population is progressive. Chronic inflammation, recurrent infections, and epithelial damage likely accumulate over time, resulting in worsening olfaction [19]. This trend underlines the importance of the early identification and monitoring of olfactory health in PCD patients. While the *RSPH4A* founder mutation is known to result in central pair apparatus defects, its phenotypic correlation with olfactory dysfunction has not been explicitly documented. Our findings suggest that patients with this mutation may have a distinct olfactory phenotype, highlighting the need for genotype–phenotype correlation studies in PCD [8]. In addition to clinical implications, our study raises important questions about the quality of life. Anosmia can negatively affect nutrition, safety, and mental health [5]. Therefore, olfactory testing should be integrated into routine care, and patients should be counseled on strategies to mitigate associated risks.

This study has several limitations. The sample size was small, limited by the rarity of the condition and the specific mutation. Although the trends observed were significant and consistent, larger cohorts are needed to validate our findings and allow for multivariate analyses. We also relied solely on the BSIT to evaluate olfactory function. While it is a validated and efficient tool, combining it with other assessments—such as threshold and discrimination testing or patient-reported outcome measures like the Questionnaire of Olfactory Disorders (QOD)—would offer a more comprehensive evaluation. Additionally, we did not control for potential confounders such as environmental exposures, medication use, or comorbidities that may impact olfaction. Longitudinal studies are needed to assess how olfactory dysfunction evolves over time and whether targeted interventions (e.g., nasal steroids, olfactory training) can alter its course. Future research should explore olfactory dysfunction across different PCD genotypes, employ imaging to evaluate structural anomalies in the olfactory clefts, and investigate the role of sex hormones in modulating disease expression.

## 5. Conclusions

Olfactory impairment is a significant and under-recognized manifestation among Puerto Rican patients with PCD carrying the *RSPH4A* founder mutation. Our findings demonstrate that both male and female PCD patients exhibit reduced olfactory function compared to healthy controls, with females showing a more pronounced and progressive age-related decline. These results highlight a potential sex-specific vulnerability that may be driven by hormonal, inflammatory, or genetic factors unique to this mutation. Given the clinical implications of olfactory dysfunction, routine screening for smell impairment should be incorporated into standard care for PCD patients. Supportive interventions such as olfactory training, the management of upper airway inflammation, and patient education on the risks associated with anosmia may help mitigate the broader impact of this sensory deficit.

## Data Availability

All data are available upon request through the corresponding author.
